# The Milk Supply of a Hospital: American Method of Sterilisation

**Published:** 1904-07-16

**Authors:** Sydney Phillips

**Affiliations:** Barrister-at-Law, Steward of St. Thomas's Hospital.


### THE MILK SUPPLY OF A HOSPITAL: AMERICAN METHOD OF STERILISATION.

By Sydney Phillips, B.A., Barrister-at-Law, Steward of St. Thomas's Hospital.

The important question of the milk supply has for some years had special attention paid to it at St. Thomas's Hospital, and as we were the first—perhaps, as yet, the only—English hospital, to adopt the Potts Pasteuriser, it is thought that some account of our methods may be of interest. The special importance of the milk question to us may be gathered from the fact that we use over 1,100 gallons per week, and it is to be remembered that the major portion of this large quantity is consumed by those patients who are in the gravest condition, namely those on fever diet.

For a satisfactory milk supply it is necessary to secure:—

1. That the milk delivered shall be genuine and free from adulteration.
2. That it shall be free from infection.
3. That it shall be sterilised and supplied to the wards in such a condition that it can be kept sweet for a maximum period of 16 hours (*i.e.*, from 4 P.M. one day to 8 A.M. the next).

We secure the first two essentials by the form of our contract. Our stipulations are:—

"Milk—genuine, new. To contain not less than 3 per cent. of butter fat, and without any added preservatives. Specific gravity 1·032. Total solids not less than 12 per cent. Solids not fat, not less than 8·5 per cent. The contractor undertakes not to deliver milk from any farm or dairy the address of which has not been notified to the hospital, and the authorities of the hospital shall be empowered to inspect such farm or dairy or call for evidence that it is in a satisfactory sanitary condition and that the cows thereon are free from tuberculous or other disease."

Besides this stipulation there are, of course, various clauses of a purely legal character, empowering the hospital under certain circumstances to purchase elsewhere at the expense of the contractor, to summarily cancel the contract, etc.

To ascertain whether the contractor is carrying out his undertaking, samples are frequently taken and submitted for examination to the consulting chemist and to a bacteriologist.

The third essential (sterilisation, etc.) is the one that principally concerns us at the present time, and it is to our American cousins that we are indebted both for the scientific research and for the mechanical apparatus necessary to secure this.

In 1900 Dr. H. L. Russell, the bacteriologist at the Agricultural Experiment Station of the University of Wisconsin, U.S.A., made some investigations on the thermal death point of the tubercle bacillus under commercial conditions. He chose the tubercle bacillus for the reason that a higher degree of heat was apparently required to destroy this than other disease-producing bacteria likely to occur in milk. The full account of his investigations is very interesting, and can be read in the "Seventeenth Annual Report (for the year ended June 30th, 1900) of the Agricultural Experiment Station of the University of Wisconsin," pages 147–170. The results of his experiments, which were confirmed by Dr. Macfadyen, of the Lister Institute, he thus summarises:—

1. An exposure of tuberculous milk in a tightly-closed commercial pasteuriser for a period of 10 minutes destroyed in every case the tubercle bacillus, as determined by the inoculation of such heated milk into susceptible animals like guinea-pigs.
2. When milk was exposed under conditions that would enable a surface pellicle or membrane to form on the surface, the tubercle organism is able to resist the action of heat at 140° F. (60° C.) for considerably longer periods of time.
3. Efficient pasteurisation can be more readily accomplished in a closed receptacle, such as is most frequently used in the commercial treatment of milk, than where the milk is heated in open bottles or open vats.
4. *It is recommended, in order to thoroughly pasteurise milk so as to destroy any tubercle bacilli which it may contain, without in any way injuring its creaming properties or consistency, to heat the same in closed pasteurisers for a period of not less than* 20 *minutes at* 140° F.

He further says that this standard had been in constant use for over a year in the University Creamery and had been abundantly confirmed.

In a form of steriliser which is largely used in England, and was at one time used at St. Thomas's, the milk is carried to a temperature of 195° F. Many disadvantages result. The milk acquires a boiled flavour, and the consistency of the milk is materially changed. The cream does not rise readily and the consistency or "body" of cream itself is much lessened. The most serious disadvantage, from a practical point of view, however, is that after exposure to this high temperature the task of cooling is very tedious and wasteful of water, and the keeping quality of the milk is consequently impaired. With this method of "sterilising" the temperature cannot be reduced below about 82° F., and that only after 50 minutes cooling. For the proper preservation of milk it is indispensable that the milk should be rapidly cooled to about 50° F. With the defective apparatus alluded to, as it was not tightly closed, and as it had no efficient method of stirring, a scum invariably formed on the surface of the milk and the destruction of bacilli was therefore uncertain.

In view of the defects of this system it was decided to abandon it and substitute the machine with which the Wisconsin University tested its investigations—viz., the Potts Pasteuriser. It is manufactured by the Creamery Package Company, Chicago, in various sizes, ranging from 50 to 250 gallons (American), and has been widely used throughout the United States during the last few years. The machine we have adopted contains 100 American gallons—that is, about 83 English gallons.

It consists of two cylinders, one within the other, with a space of about one inch separating the two. The inner cylinder is made of tinned copper, whilst the outer one is of steel, jacketed with wood. The space between the two cylinders is first filled with water and the milk to be pasteurised is poured into the inner drum, which is tightly closed. Steam is then turned into the water space to heat the milk to the required temperature, while by means of a four h.-p. electric motor the machine is revolved at the rate of from 10 to 15 revolutions per minute. There is a wing attachment on the inner side of the inner cylinder which aids in agitating the milk as the machine revolves, so that the temperature of the milk rises uniformly. Thus the milk is kept thoroughly mixed and formation of film is prevented. About 13 minutes are required to bring the temperature of the milk to 140° F., at which it is held for 30 minutes. The steam is then turned off and cold water is admitted into the space between the two cylinders. After 25 minutes the temperature is reduced to 86° F. Brine, which has been cooled by mechanical refrigeration to a temperature of 16° F., is then admitted, and after about 20 minutes the temperature is reduced to 48° F.

The combination of a heater and cooler in one is very convenient, and from the beginning to the end of the process the milk is unexposed to the air. The milk seems to be improved by the treatment; and in summer weather it is very delicious to drink milk at this unusually cool temperature. The process is very essential for keeping milk. One summer 65 observations of sterilised and unsterilised milk were made during a month of hot weather. The sterilised milk was always found to be sweet 16 hours after delivery, whereas the unsterilised was found to be sour on 26 occasions.

